# Association of Serum Albumin Levels With Preeclampsia Severity and Fetomaternal Outcomes: A Cross-Sectional Study

**DOI:** 10.7759/cureus.114147

**Published:** 2026-08-07

**Authors:** Laxmi Anjenappa, Mahesh Venkatachari

**Affiliations:** 1 Obstetrics and Gynecology, All India Institute of Medical Sciences, Mangalagiri, Mangalagiri, IND; 2 Obstetrics and Gynecology, Lady Hardinge Medical College, New Delhi, IND; 3 Pediatric Nephrology, All India Institute of Medical Sciences, Mangalagiri, Mangalagiri, IND

**Keywords:** hypoalbuminaemia, maternal outcome, neonatal outcome, preeclampsia, serum albumin, severe features

## Abstract

Background: Serum albumin is frequently reduced in preeclampsia because of urinary protein loss, endothelial dysfunction, capillary leakage, and pregnancy-related haemodilution. Its value as an independent marker of severe disease or adverse pregnancy outcomes remains uncertain.

Methods: This hospital-based observational cross-sectional study included 150 women with singleton pregnancies and preeclampsia. The source report classified 99 women as having preeclampsia without severe features and 51 as having preeclampsia with severe features. Serum albumin was the primary biochemical parameter and was grouped as less than 2.5, 2.5 to less than 3.0, and 3.0 to 3.5 g/dL. Urine protein by dipstick, spot urine protein-to-creatinine ratio, and 24-hour urinary protein were also recorded as supportive renal measures. Maternal and perinatal outcomes were compared using chi-square or Fisher exact tests, with unadjusted risk ratios and 95% confidence intervals.

Results: The mean serum albumin concentration was 3.08 ± 0.27 g/dL, and no participant had a value above 3.5 g/dL. Albumin below 3.0 g/dL was reported in 25 of 51 women (49.0%) with severe features and 24 of 99 (24.2%) without severe features (risk ratio 2.02, 95% confidence interval 1.29-3.16; p=0.003). Urine dipstick proteinuria of 3+ or 4+ was more frequent in women with severe features (27.5% versus 9.1%; risk ratio 3.02, 95% confidence interval 1.40-6.50; p=0.007). Severe features were also associated with preterm delivery before 37 weeks, neonatal intensive care admission, birth weight below 2 kg, and impending eclampsia. Caesarean delivery, a five-minute Apgar score below seven, and perinatal death did not differ significantly.

Conclusions: The absence of a higher-albumin comparator limited assessment of the prespecified 3.5 g/dL threshold. Albumin below 3.0 g/dL was associated with severe features in an exploratory unadjusted analysis, but an independent prognostic value was not established. Serum albumin should be interpreted as an adjunct to established clinical and laboratory severity criteria.

## Introduction

Preeclampsia is a multisystem pregnancy disorder characterised by new-onset hypertension after 20 weeks of gestation with proteinuria and/or maternal organ or uteroplacental dysfunction. It remains an important contributor to maternal morbidity, medically indicated preterm birth, and perinatal morbidity. Contemporary guidance classifies disease according to the presence of severe features rather than using the terms mild and severe preeclampsia [1,2].

Serum albumin concentration decreases physiologically during pregnancy because of plasma-volume expansion and altered renal handling of protein [3,4]. In preeclampsia, urinary protein loss, glomerular endothelial injury, increased capillary permeability, and altered hepatic synthesis may cause a further reduction in circulating albumin [3-5]. Albumin is inexpensive and routinely measured, making it an attractive potential marker in resource-constrained settings. However, it is non-specific and is influenced by gestational age, nutrition, hepatic function, inflammation, and renal protein loss. Consequently, a low value may reflect overall disease burden without independently predicting clinically important outcomes.

Previous observational studies have reported lower albumin or total protein concentrations in women with severe preeclampsia and associations with earlier delivery, severe hypertension, foetal growth restriction, neonatal intensive care admission, ascites, and maternal organ dysfunction [6-9]. Studies examining proteinuria and hypoproteinaemia have also linked more marked biochemical abnormalities with pulmonary oedema, preterm delivery, and adverse neonatal outcomes, although the strength and independence of these relationships have varied [10-13]. More recent albumin-focused studies have suggested cut-offs between approximately 2.3 and 2.5 g/dL for identifying severe features, but adjusted analyses have not consistently shown albumin to be an independent predictor [14,15]. The clinically useful threshold and incremental value beyond standard diagnostic criteria therefore remain uncertain.

Despite the biological plausibility and routine availability of serum albumin, uncertainty remains regarding the clinically meaningful threshold and whether albumin provides information beyond established clinical and laboratory markers of preeclampsia severity. This uncertainty supports further evaluation of albumin as an adjunctive, hypothesis-generating marker rather than as a stand-alone prognostic test.

## Materials and methods

Study design and setting

This was a hospital-based observational cross-sectional study conducted in the Department of Obstetrics and Gynaecology at a tertiary care center in North India. Women were recruited from the labour room from January 1, 2021, through December 31, 2021 (12 months) and were observed through delivery and the immediate postnatal period.

Participants

Women with a singleton pregnancy diagnosed with preeclampsia, irrespective of age, parity, or gestational age, were eligible after providing written informed consent. Exclusion criteria were hypertension predating pregnancy or another chronic hypertensive disorder, known kidney or liver disease, severe anaemia, autoimmune disease, multiple pregnancy, and diabetes mellitus. Eligible consenting women were recruited consecutively from the labour room during the study period. The archived study records did not contain a complete screening log; therefore, the total number screened, the number who declined participation, and whether recruitment was strictly consecutive could not be reconstructed. No enrolled participant was excluded from the analytical cohort because of an unavailable serum albumin measurement; serum albumin data were available for all 150 enrolled women.

Study objectives

The primary objective was to evaluate the association between maternal serum albumin concentration and the severity of preeclampsia. The secondary objective was to evaluate the association between maternal serum albumin concentration and adverse maternal and perinatal outcomes. The later comparison of albumin below 3.0 g/dL with 3.0-3.5 g/dL was not a prespecified objective and was treated as an exploratory post hoc analysis.

Sample size and recruitment

The original study protocol estimated a sample size of 256, assuming an 86.2% prevalence of hypoalbuminaemia in preeclampsia and an allowable error of 5% of the assumed prevalence. The protocol subsequently proposed a sample size of 150 participants due to logistic issues; this is acknowledged as a limitation.

Clinical classification

The original protocol classified participants as mild preeclampsia (Group A) or severe preeclampsia (Group B) using contemporaneous departmental criteria. For current terminology, these groups are reported as preeclampsia without severe features and preeclampsia with severe features, respectively, in accordance with contemporary classification frameworks [1,2]. Severe hypertension and evidence of clinically important end-organ dysfunction were considered severe features. All women received management according to institutional protocols.

Laboratory assessment

A detailed history and examination were completed. Routine evaluation included complete blood count, platelet count, liver and renal function tests, urine protein assessment, and serum albumin. Renal protein assessment included semiquantitative urine protein by dipstick, spot urine protein-to-creatinine ratio (UPCR), and 24-hour urinary protein. Serum albumin remained the primary biochemical parameter of interest, while proteinuria measurements were considered supportive secondary renal markers. For biochemical testing, 2 mL of venous blood was allowed to clot, centrifuged at 4500 rpm for five minutes, and the serum was stored at -20 degrees C until analysis on a Beckman Coulter analyser (Beckman Coulter Inc., Brea, California, USA). The protocol defined hypoalbuminaemia as serum albumin below 3.5 g/dL. Albumin was categorised as less than 2.5, 2.5 to less than 3.0, and 3.0 to 3.5 g/dL.

Outcomes

Maternal outcomes included impending eclampsia, eclampsia, pulmonary oedema, haemolysis, elevated liver enzymes and low platelet count syndrome, puerperal sepsis, mode of delivery, and postnatal complications. Perinatal outcomes included gestational age at delivery, birth weight, five-minute Apgar score, neonatal intensive care unit admission, and perinatal death.

Statistical analysis

Continuous data are presented as mean ± standard deviation when supported by the source dataset, and categorical data as number and percentage. The source protocol specified Student t-test for normally distributed data, Mann-Whitney U test for skewed data, and a two-sided significance level of 0.05. For manuscript preparation, aggregate cell counts reported in the thesis were additionally analysed using Pearson chi-square or Fisher exact tests, as appropriate. Risk ratios and 95% confidence intervals were calculated for binary outcomes. The protocol-defined threshold for hypoalbuminaemia was less than 3.5 g/dL. Because no participant had a serum albumin value above 3.5 g/dL, an exploratory post hoc comparison of less than 3.0 g/dL versus 3.0-3.5 g/dL was undertaken. The 3.0 g/dL threshold was literature-informed, including prior studies that evaluated albumin around this level, but it was not prespecified and should not be interpreted as a validated diagnostic threshold. Although individual-level clinical and laboratory data were available, the archived dataset did not contain a definitive patient-level severity-group variable corresponding exactly to the original 99/51 source classification. Retrospective reconstruction of severity from blood pressure, symptoms, free-text diagnoses, and laboratory values could introduce misclassification; therefore, multivariable adjustment using the source-defined severity groups was not performed. The reported associations are consequently unadjusted and susceptible to residual confounding.

Ethical considerations

The Institutional Ethics Committee of Lady Hardinge Medical College and Associated Hospitals, New Delhi, approved the study (LHMC/IEC/2020/PG Thesis/80; December 29, 2020). Written informed consent was obtained from all participants. The study involved evaluation and observation, and identifying information was not used in the analysis or manuscript.

## Results

Among 150 women with preeclampsia, the source report classified 99 (66.0%) as having preeclampsia without severe features and 51 (34.0%) as having preeclampsia with severe features. The mean age was 27.42 ± 4.20 years, the mean body mass index was 23.37 ± 2.12 kg/m^2^, and the mean serum albumin concentration was 3.08 ± 0.27 g/dL. Group-specific age, body mass index, serum albumin, gestational age, and blood-pressure characteristics are summarised in Table 1. No participant had a reported serum albumin concentration above 3.5 g/dL.

**Table 1:** Distribution of key clinical characteristics according to preeclampsia severity Continuous variables are presented as mean ± standard deviation and were compared using the independent-samples Student’s t-test. Categorical variables are presented as number (percentage) and were compared using Pearson’s chi-square test. A two-sided p-value <0.05 was considered statistically significant.

Characteristic	Preeclampsia without severe features (n=99)	Preeclampsia with severe features (n=51)	Test statistic	p-value
Age, years, mean ± SD	27.51 ± 4.17	27.25 ± 4.34	t = 0.344	0.732
BMI, kg/m², mean ± SD	23.41 ± 2.15	23.31 ± 2.10	t = 0.289	0.773
Serum albumin, g/dL, mean ± SD	3.15 ± 0.22	2.96 ± 0.31	t = 4.394	<0.001
Gestational age at delivery, weeks, n (%)			χ² = 18.914	<0.001
28–30 weeks	3 (3.0)	3 (5.9)		
31–33 weeks	2 (2.0)	11 (21.6)		
34–36 weeks	12 (12.1)	8 (15.7)		
≥37 weeks	82 (82.8)	29 (56.9)		
Serum albumin category, n (%)			χ² = 9.840	0.007
<2.5 g/dL	2 (2.0)	1 (2.0)		
2.5–<3.0 g/dL	22 (22.2)	24 (47.1)		
3.0–3.5 g/dL	75 (75.8)	26 (51.0)		
Systolic blood pressure ≥160 mmHg, n (%)	0 (0)	32 (62.7)	χ² = 78.963	<0.001
Diastolic blood pressure ≥110 mmHg, n (%)	0 (0)	24 (47.1)	χ² = 55.462	<0.001

Albumin concentrations differed across the reported severity groups (overall three-category chi-square p=0.007). Albumin below 3.0 g/dL was reported in 25 of 51 women (49.0%) with severe features and 24 of 99 (24.2%) without severe features (risk ratio 2.02, 95% confidence interval 1.29-3.16; Fisher exact p=0.003) (Table 2). The corresponding unadjusted odds ratio was 3.00 (95% confidence interval 1.47-6.15). The lower albumin category contained few participants and was unsuitable for a reliable threshold analysis.

**Table 2:** Association between serum albumin strata and preeclampsia severity Data are presented as number (percentage). The risk ratio compares the proportion of women with serum albumin <3.0 g/dL among those with severe features with the corresponding proportion among women without severe features. A two-sided p-value <0.05 was considered statistically significant.

Albumin stratum	Without severe features (n=99)	With severe features (n=51)	Unadjusted effect	95% CI	p-value
<3.0 g/dL	24 (24.2%)	25 (49.0%)	RR 2.02	1.29–3.16	0.003
3.0–<3.5 g/dL	75 (75.8%)	26 (51.0%)	Reference	—	—

Proteinuria was also assessed as a supportive renal marker. On urine dipstick testing, 1+ proteinuria was present in 26/99 (26.3%) women without severe features and 7/51 (13.7%) with severe features; 2+ in 64/99 (64.6%) and 30/51 (58.8%); 3+ in 8/99 (8.1%) and 10/51 (19.6%); and 4+ in 1/99 (1.0%) and 4/51 (7.8%), respectively. The overall dipstick distribution differed between severity groups (chi-square=11.03, df=3; p=0.012). Proteinuria of at least 3+ was more frequent in women with severe features (27.5% versus 9.1%; risk ratio 3.02, 95% confidence interval 1.40-6.50; Fisher exact p=0.007).

Women with severe features delivered earlier and had less favourable neonatal outcomes. Preterm delivery before 37 weeks occurred in 22 of 51 women (43.1%) with severe features and 17 of 99 (17.2%) without severe features (risk ratio 2.51, 95% confidence interval 1.47-4.29; p<0.001). Neonatal intensive care admission occurred in 25 of 51 (49.0%) versus nine of 99 neonates (9.1%) (risk ratio 5.39, 95% confidence interval 2.72-10.67; p<0.001), and birth weight below 2 kg occurred in 21 of 51 (41.2%) versus 13 of 99 (13.1%) (risk ratio 3.14, 95% confidence interval 1.71-5.73; p<0.001).

Impending eclampsia was more frequent among women with severe features (29.4% versus 7.1%; risk ratio 4.16, 95% confidence interval 1.81-9.55; p<0.001). Differences in eclampsia, pulmonary oedema, caesarean delivery, five-minute APGAR score below seven, and perinatal death were not statistically significant. No maternal death was reported. Maternal and perinatal outcomes are summarised in Table 3.

**Table 3:** Maternal and perinatal outcomes according to preeclampsia severity Data are presented as number (percentage). The total sample sizes are provided in the column headings and are not repeated within individual cells. Risk ratios are unadjusted and compare women with severe features with women without severe features. CI, confidence interval; NICU, neonatal intensive care unit; RR, risk ratio. A two-sided p-value <0.05 was considered statistically significant.

Outcome	Without severe features (n=99)	With severe features (n=51)	RR	95% CI	p-value
Impending eclampsia	7 (7.1%)	15 (29.4%)	4.16	1.81–9.55	<0.001
Eclampsia	3 (3.0%)	6 (11.8%)	3.88	1.01–14.89	0.062
Pulmonary oedema	1 (1.0%)	2 (3.9%)	3.88	0.36–41.81	0.267
Caesarean delivery	43 (43.4%)	25 (49.0%)	1.13	0.79–1.62	0.604
Preterm delivery <37 weeks	17 (17.2%)	22 (43.1%)	2.51	1.47–4.29	<0.001
Birth weight <2 kg	13 (13.1%)	21 (41.2%)	3.14	1.71–5.73	<0.001
NICU admission	9 (9.1%)	25 (49.0%)	5.39	2.72–10.67	<0.001
Five-minute Apgar score <7	6 (6.1%)	4 (7.8%)	1.29	0.38–4.38	0.735
Perinatal death	5 (5.1%)	6 (11.8%)	2.33	0.75–7.27	0.185
Any postnatal complication	5 (5.1%)	9 (17.6%)	3.49	1.24–9.88	0.017

## Discussion

The principal finding was that the observed albumin distribution did not include a value above 3.5 g/dL and therefore could not adequately evaluate the prespecified threshold as a binary discriminator. Within the observed range, albumin below 3.0 g/dL was approximately twice as prevalent among women with severe features. This 3.0 g/dL comparison was literature-informed but post hoc, exploratory, and unadjusted. The study therefore supports a possible gradient between lower albumin and overall disease burden but does not establish serum albumin as an independent prognostic test or a basis for clinical guideline formulation.

The observed pattern is biologically plausible. In preeclampsia, glomerular endothelial injury increases urinary protein loss, while systemic endothelial dysfunction and capillary leakage redistribute albumin into the interstitial space [3-5]. Reduced effective circulating volume and altered hepatic synthesis may contribute further. Nevertheless, pregnancy-related haemodilution, nutritional status, inflammation, and the timing of sampling also influence serum albumin. A threshold that is useful outside pregnancy may therefore be insufficiently specific in late gestation.

Chen et al. reported earlier delivery, more severe hypertension, organ dysfunction, ascites, abruption, caesarean birth, fetal growth restriction, and neonatal intensive care admission among women with severe hypoproteinaemia [6]. Benoit and Rey found lower albumin concentrations in severe preeclampsia, but albumin below 20 g/L was not an independent severity criterion because affected women already had other adverse conditions [7]. Gojnic et al. reported that all women with severe disease in their cohort had albumin below 3.0 g/dL [8], while Guan et al. linked hypoproteinaemia with ascites during expectant management of early-onset severe preeclampsia [9]. In the present cohort, women with severe features similarly had higher risks of preterm birth, low birth weight, and neonatal intensive care admission. These neonatal outcomes cannot be attributed to low albumin itself because the analysis did not adjust for gestational age and disease severity.

Recent evidence remains mixed regarding clinically useful cut-offs. Ciciu et al. found lower albumin in severe disease and identified a 2.3 g/dL threshold with reasonable discrimination, but albumin below 2 g/dL was not independently associated with severe preeclampsia after adjustment and did not predict most maternal or fetal complications [14]. Conversely, a 2026 Indonesian multicentre study reported that severe hypoalbuminaemia was associated with a 4.4-fold higher risk of severe features and proposed a cut-off of 2.51 g/dL [15]. Differences in case mix, timing of sampling, albumin assays, definitions, and adjustment strategies may account for the divergent results. Together, these studies favour evaluating lower, pregnancy-specific thresholds rather than treating 3.5 g/dL as a universal binary cut-off.

The high frequency of impending eclampsia, preterm birth, and neonatal intensive care admission in the severe-feature group underscores the importance of standard clinical and laboratory surveillance. Proteinuria was not omitted from the source evaluation: urine dipstick protein, spot UPCR, and 24-hour urinary protein were recorded, and higher-grade dipstick proteinuria was more frequent among women with severe features in the present reanalysis. Studies of proteinuria have reported conflicting prognostic performance: some cohorts found associations with adverse maternal and neonatal outcomes [10,12,13], whereas a large multicentre study suggested that severe proteinuria and associated hypoalbuminaemia were more closely related to pulmonary oedema or pleural effusion than to severe hypertension itself [11]. Albumin may therefore add contextual information, particularly when markedly reduced or accompanied by heavy proteinuria, ascites, or pulmonary oedema, but it should not replace established severe-feature criteria. Broader biomarker studies similarly show that biochemical abnormalities can correlate with complications yet have limited stand-alone predictive utility [16-18].

The association between low albumin and disease severity should not be interpreted as evidence that albumin infusion improves outcomes. Small clinical studies found that repeated albumin administration did not produce sustained reductions in blood pressure or renal improvement and did not consistently improve intervillous blood flow [19,20].

This study has several limitations. It was conducted at a single tertiary-care centre and had a small sample size. Rare maternal complications were infrequent, and the unadjusted estimates are imprecise for uncommon outcomes. The albumin distribution was narrow, and the absence of a value above 3.5 g/dL limited evaluation of the prespecified binary threshold. The less-than-3.0 g/dL comparison was post hoc and should be considered hypothesis-generating rather than a validated clinical cut-off. Albumin and disease severity were assessed during the same admission, limiting temporal interpretation and precluding causal or prospective predictive conclusions. Residual confounding by gestational age, magnitude of proteinuria, nutritional status, hepatic and renal dysfunction, and overall disease severity may therefore explain part of the observed associations. Recruitment at delivery, the tertiary-referral setting, and exclusion of several major comorbidities further limit applicability to the broader population of women with preeclampsia.

Strengths include assessment of an inexpensive, routinely available biochemical parameter together with urine protein measures; inclusion of both maternal and neonatal outcomes; and observation through the immediate postpartum period. The findings should be considered exploratory and hypothesis-generating. Future studies should use prospective multicentre recruitment, a documented screening flow, the planned sample size, prespecified pregnancy-specific albumin thresholds, standardized albumin assay methodology and sampling before major treatment or delivery, concurrent quantification of proteinuria, and multivariable models with internal validation.

## Conclusions

Lower serum albumin concentrations may be associated with greater disease severity in women with preeclampsia; however, serum albumin alone cannot be considered an independent predictor of severity or adverse fetomaternal outcomes. It should therefore be interpreted as an adjunct to established clinical and laboratory severity criteria. Prospective multicentre studies using predefined pregnancy-specific thresholds, clearly timed measurements, and multivariable validation are required before serum albumin can be incorporated into severity prediction.
